# Extracellular Membrane Vesicles as Vehicles for Brain Cell-to-Cell Interactions in Physiological as well as Pathological Conditions

**DOI:** 10.1155/2015/152926

**Published:** 2015-10-25

**Authors:** Gabriella Schiera, Carlo Maria Di Liegro, Italia Di Liegro

**Affiliations:** ^1^Dipartimento di Scienze e Tecnologie Biologiche, Chimiche e Farmaceutiche (STEBICEF), Università degli Studi di Palermo, 90128 Palermo, Italy; ^2^Dipartimento di Biomedicina Sperimentale e Neuroscienze Cliniche (BIONEC), Università degli Studi di Palermo, 90127 Palermo, Italy

## Abstract

Extracellular vesicles are involved in a great variety of physiological events occurring in the nervous system, such as cross talk among neurons and glial cells in synapse development and function, integrated neuronal plasticity, neuronal-glial metabolic exchanges, and synthesis and dynamic renewal of myelin. Many of these EV-mediated processes depend on the exchange of proteins, mRNAs, and noncoding RNAs, including miRNAs, which occurs among glial and neuronal cells. In addition, production and exchange of EVs can be modified under pathological conditions, such as brain cancer and neurodegeneration. Like other cancer cells, brain tumours can use EVs to secrete factors, which allow escaping from immune surveillance, and to transfer molecules into the surrounding cells, thus transforming their phenotype. Moreover, EVs can function as a way to discard material dangerous to cancer cells, such as differentiation-inducing proteins, and even drugs. Intriguingly, EVs seem to be also involved in spreading through the brain of aggregated proteins, such as prions and aggregated tau protein. Finally, EVs can carry useful biomarkers for the early diagnosis of diseases. Herein we summarize possible roles of EVs in brain physiological functions and discuss their involvement in the horizontal spreading, from cell to cell, of both cancer and neurodegenerative pathologies.

## 1. Introduction

Extracellular vesicles (EVs) are membrane structures that can be divided into two subgroups: membrane vesicles (MVs), also named ectosomes [1], that derive from plasma membrane exocytosis and have dimensions in the range of 100 nm–1 *μ*m and exosomes that are smaller vesicles of 50–100 nm in diameter, generated by exocytosis of multivesicular bodies (MVBs) [2]. The two classes of EVs share membrane components, but each of them also contains peculiar proteins, some of which are cell-specific [1]. Lipid composition is also peculiar; MVBs are enriched with specific lipids, probably involved in membrane budding, such as cholesterol, the unconventional phospholipid lysobisphosphatidic acid, sphingomyelins, and ceramide [1, 3–6]. Similarly, cholesterol, sphingomyelin, and ceramide also segregate during ectosomes formation at the plasma membrane [7]. It has been suggested that sorting of proteins to ectosomes is largely based on their interaction with the membrane through their lipid anchors which concentrate them to specific plasma membrane microdomains [8, 9].

Specific enrichment of both proteins and lipids in EVs not only suggests the existence of underlying regulatory mechanisms, which allow selection of cargos, but also points to an important role of EVs in the normal tissue physiology. As a confirmation of such a central role, it is now clear that vesicle release is a universal phenomenon, representing a novel and significant way to shuttle different molecules, such as protein, DNA, and RNA among cells, not only in eukaryotic organisms, but also in prokaryotes [10, 11].

Moreover, although vesicle release was initially discovered in tumor cells [12–16], vesicles are clearly produced by several nontumor cells [17–21] and can reach most biological fluids, such as blood plasma [22], synovial fluids [23, 24], breast milk [25], and saliva [26, 27], where, in addition, their concentration can dramatically change under pathological conditions.

Although EVs have been finally accepted as significant vehicles for cell-to-cell communication and they have been identified in a variety of organisms and conditions, two main problems remain, however, unsolved:

1. the actual role of these vesicles in the integrated behavior of tissues and organs;
2. the reason why production of EVs and specific sorting of cargos to them are altered in pathological conditions as different as cancer and neurodegeneration.

Moreover, since most studies have been performed on EV mixtures, it is still unclear whether these different classes of vesicles are equally involved in physiological and pathological processes.

Here we summarize the possible physiological roles of EVs in the nervous system and discuss their involvement in the horizontal transfer of brain pathologies.

## 2. Vesicles in Brain Normal Cells

Cell-to-cell communication is determinant for the right mammalian brain maturation to regulate differentiation of neurons, endothelial and glial cells, as well as allow formation and stabilization of synapses. Cross talk among different classes of brain cells is also essential to generate the blood-brain barrier (BBB), which then maintains brain internal milieu, by controlling trafficking of molecules and ions between the brain and the blood [28]. For example, in a transwell coculture system, containing both rat cortical astrocytes and neurons, brain capillary endothelial cells (BCECs) were found to form over time a functional barrier layer, even in the absence of cell-to-cell contacts [29, 30]. Further analyses demonstrated that both neurons [19] and astrocytes [20] release into the medium, at least in part by EVs, Vascular Endothelial Growth Factor (VEGF) and Fibroblast Growth Factor 2 (FGF-2; also known as basic FGF), two growth factors which promote vascularization of developing brain. Both factors are also involved in abnormal neovascularization processes which accompany several pathological conditions [31, 32]. Interestingly, FGF-2 has been known since a long time ago to be secreted in spite of lacking a conventional secretion sequence and independently of the classical cell secretion pathway [33].

**Figure 1:**
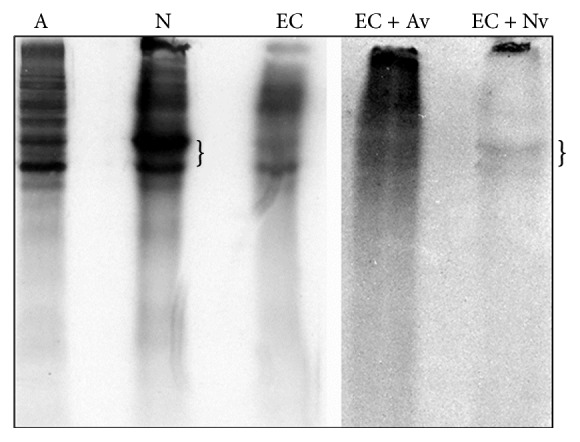
Fluorography of total cell lysates from astrocytes (A), neurons (N), and endothelial cells (EC), metabolically labeled with ^35^S-methionine, as well as from unlabeled endothelial cells, incubated for 24 hours with microvesicles shed from labeled astrocytes (EC + Av) or neurons (EC + Nv). The brackets indicate bands present in both labeled neurons and endothelial cells incubated with vesicles shed from neurons.

One of the main problems in studying metabolism of EVs has been to understand how their content reaches target cells. There is now evidence that their cargos can be at least partially transferred into the recipient cells, through fusion of EVs to the target plasma membranes. For example, after being released via EVs from metabolically labeled astrocytes/neurons, radioactive proteins have been found in unlabeled endothelial cells (Figure 1).

As mentioned, extracellular vesicles also contain mRNAs and different species of noncoding RNA. Given the importance of RNA-binding proteins (RBPs) in posttranscriptional regulation, mRNAs in vesicles are probably bound to proteins in the form of ribonucleoprotein particles. In agreement with this hypothesis, a microarray analysis, performed on vesicles purified from the cerebrospinal fluid (CSF), recently demonstrated the presence of RNAs containing the recognition sequence for hnRNPA2/B1, an RBP present in the same vesicles, probably in sumoylated form. Moreover, sumoylation has been shown to control hnRNPA2/B1 binding to miRNAs and sorting of these RNAs to exosomes [34]. Both the amount of vesicles and the number of mRNA molecules which bear the hnRNPA2/B1 recognition sequence decrease with development [35].

When we consider the complexity of vesicle cargos, it is not surprising that EVs also contain molecular chaperones and other factors which can regulate protein folding as well as protein-protein and protein-nucleic acid interactions. MVs and exosomes released from astrocytes not only contain, for example, Hsc70/Hsp70 [36, 37] and synapsin I [38], but also matrix metalloproteinases [39], which could be involved in extracellular matrix remodeling. This latter ability is fundamental for tumor cells invasion and migration; however, which function could it have in normal astrocytes or neurons? To answer the question, it could be useful to consider that most brain cells are able to grow branched cellular processes, which can explore the environment, migrate, and contact other cells; thus, an intrinsically high ability to modify the surrounding environment is critical for these cells.

Interestingly,* in vitro* studies demonstrated that release of exosomes from neurons can be modulated by synaptic activity [40]; by functioning as vehicles for both anterograde and retrograde information transfer, exosomes could be then involved in synaptic plasticity and long-term memory [41].

Vesicles are also released from oligodendrocytes, the glial cells responsible in the CNS for producing the myelin sheath which coats the axons, allowing fast impulse conduction; in addition, like astrocytes, oligodendrocytes have a trophic function and provide neurons with energetic substrates, such as lactate [42–44]. The continuous axon-oligodendrocyte cross talk seems to be mostly based on transfer of vesicles [42] which contain myelin proteins, such as proteolipid protein (PLP), 2′3′-cyclic-nucleotide 3′-phosphodiesterase (CNP), myelin-associated glycoprotein (MAG), myelin oligodendrocyte glycoprotein (MOG), NAD-dependent deacetylase sirtuin-2, glycolytic enzymes, heat-shock proteins, and tetraspanins [45]. It has been also reported that proximal segments of transected sciatic nerves accumulate newly synthesized RNA in axons and that these mRNAs are actually synthesized in Schwann cells and then transferred to neurons through a mechanism that requires actin cytoskeleton and myosin-Va [46].

**Figure 2:**
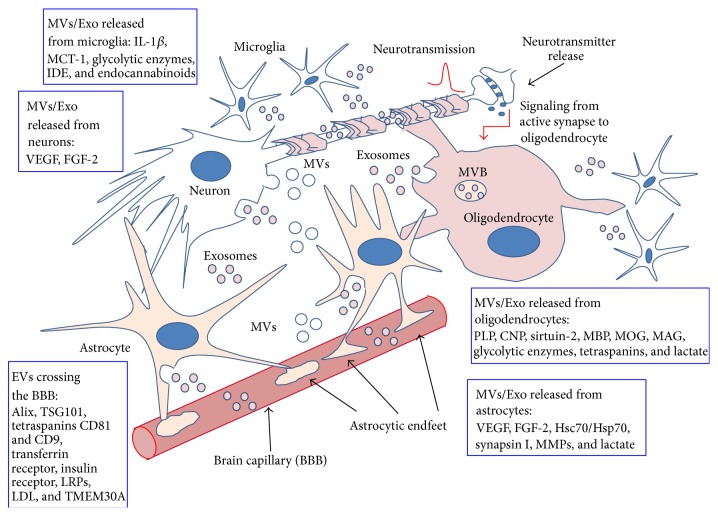
Extracellular membrane vesicles as vehicles for brain cell-to-cell interactions. As shown, all kinds of brain cells can both produce EVs and receive those produced by surrounding cells; this continuous exchange could be a fundamental source of metabolic coupling among neurons and glial cells. Vesicle trafficking from glial cells to neurons has been also suggested to be regulated by neurotransmission, as indicated by the red arrow in the figure. A few examples of molecules present in EVs released from the various brain cell types are given in the boxes. More details and related references are given in the text.

Most important, vesicle trafficking from glial cells to neurons has been suggested to be regulated by neurotransmission (Figure 2): an increase of cytosolic Ca^2+^ levels in oligodendrocytes, due to activation of glutamate receptors, present on glial cell membrane, induces exosome release [47]. Actually, active neurons should ask oligodendrocytes for metabolites, regulatory proteins, glycolytic enzymes, mRNAs, and miRNAs [48].

Transfer of mRNAs from glial cells to neurons might be of special interest when we consider that localized axonal synthesis may allow remodeling of growing (or regenerating) axons during progression through their extracellular environment. Although translation of localized mRNAs in axons has been debated for a long time [49], periaxoplasmic ribosomal plaques (PARPs) have been only recently described, which contain ribosomes attached to a plaque-like structure, also enriched with *β*-actin mRNA, molecular motors, and RNA-binding proteins [50]. Moreover, multiple translation components, including ribosomal subunits and initiation factors, interact with the transmembrane receptor (DCC) for netrin-1, suggesting that their activity can be regulated by extracellular signals [51].

The exciting possibility that ribosomes and mRNAs could be horizontally delivered from surrounding glial cells to axons has been also proposed [52, 53]. For example, Court and colleagues showed that GFP-tagged polyribosomes produced by glial cells can be transferred to axons both* in vitro* [52] and* in vivo* [54].

These findings support the idea that glial cells may contribute to local axonal protein synthesis by supplying protein synthetic machinery and specific mRNAs [55].

Another important class of brain cells is constituted by microglia, the resident macrophages of the brain, which provide the defense during infection and brain injury, and are implicated also in tissue repair. During disease, microglia acquire an activated phenotype, and release soluble mediators, to induce and maintain the inflammatory response. There is also evidence indicating that reactive microglia have the capability to release vesicles of irregular shape and size, characterized by high levels of externalized phosphatidylserine (PS) [56]. These vesicles contain IL-1*β* that may induce and propagate inflammatory reactions in the brain [56, 57]. In addition, microglial MVs, like other glial cell types (see above), are able to modulate synaptic activity and neurotransmission [58]. For example, EVs secreted by microglia have been recently shown to expose on their plasma membrane the active endocannabinoid N-arachidonoylethanolamine (AEA), which binds to and stimulates the type 1 cannabinoid receptors (CB1), thus inhibiting presynaptic transmission in GABAergic neurons [59]. Exosomes released by microglia also contain glycolytic enzymes and the monocarboxylate transporter 1 (MCT1); one role of these exosomes could be delivering to not only target cells energy substrates, but also special enzymes such as the insulin degrading enzyme (IDE), which can degrade the A*β* peptide [60].

Finally, it has been found that BCECs, the endothelial cells which constitute the wall of the brain capillaries and give rise to the blood-brain barrier, are also able to release vesicles. Interestingly, the endothelial cell-derived EVs are able to cross the BBB and are responsible, at least in part, for the brain-specific biomarkers found in blood; accordingly, these vesicles can be useful to analyze the time course of brain diseases. Since their membranes contain many BBB receptors, such as the transferrin and the insulin receptors, these MVs might be also used to deliver drugs across the BBB [61].

Now, if all the brain cell populations are able to release vesicles, an intriguing point is whether EVs produced by a given cell type have promiscuous activities or, on the contrary, have a specific target. Recent analyses suggested that physiological brain vesicles delivery is actually highly specific: exosomes secreted from stimulated glutamatergic cortical neurons were indeed captured only by other neurons; on the other hand, vesicles released from neuroblastoma cells lost this capability and were shown to bind both neurons and glial cells, with an apparent preference for glial cells [62].

Before concluding this brief summary concerning normal brain cells, it should be underlined that, beside their role as carriers of regulatory molecules, EVs can also function as scavengers. Some authors indeed found that exosomes/MVs are necessary for normal cells to eliminate proteins, for example, to discard an excess of glutamate receptor 2 [40].

## 3. Brain Cancer Cells

Variety and complexity of primary tumours of the Central Nervous System (CNS) are probably the highest among human cancers. Their classification is based on both the cell type and/or the brain structure from which they arise and on their grade, from I to IV, according to increasing malignancy of the cancer cells [63, 64].

Most brain primary tumours in the adult originate from glial cells/glial cell precursors and are collectively called gliomas, further divided into astrocytomas, oligodendrogliomas, ependymomas, and mixed oligoastrocytomas [63, 64]. According to the grade of malignancy, oligodendrocytomas and mixed gliomas are grades II and III, while astrocytomas are grouped into low-grade (LGA: pilocytic, grade I; diffuse, grade II) and high-grade astrocytomas (HGA: anaplastic, grade III; glioblastoma multiforme, GBM, grade IV) [65].

The treatment of HGA is mainly done by surgery, also required for definitive histopathologic diagnosis [65, 66], followed by radiation and chemotherapy [67].

Unfortunately, these therapeutic protocols, in spite of undisputed advancements, are not actually effective, and high grade gliomas still remain almost always fatal. In addition, gliomas are difficult to diagnose at an early stage because, at the beginning, the patients may suffer from unspecific symptoms, such as headache and seizures [65]. Difficulties in diagnosing as well as in treating gliomas also depend on the particular location of these tumours, which are protected by the BBB. Many efforts have been, indeed, recently devoted to find out strategies which might improve permeability of BBB to CNS-directed drugs (for review see [68]), although, paradoxically, BBB leakage and the concomitant vasogenic edema (see below) are the main clinical problems in patients suffering from glioblastoma (for review see [69]). For all these reasons, new approaches are urgently needed for an early diagnosis of brain cancer and improvement of therapy.

As shown for many other tumours, brain cancers release much higher amounts of extracellular vesicles than normal cells. EVs are continuously released by cancer cells, but their concentration in the body fluids is somehow proportional to disease grade [70]. Concerning their content, beside proteins more or less present in all the EVs studied up to now, such as different classes of chaperones, they contain tumour-specific antigens, MHC I and II complexes for antigen presentation, apoptosis-inducing (such as FasL and TRAIL) and immune suppressive (such as TGF*β*) factors (see below), as well as oncogenic growth factor receptors, such as a truncated form of the epidermal growth factor receptor EGFRvIII [71, 72]. In line with these contents, it has been shown that EVs shed from tumours can facilitate cancer development by suppressing immune responses, stimulating tumour growth, invasion, angiogenesis, and metastasis.

For many decades CNS was considered somehow “invisible” to the immune system because of its protected condition, ensured by BBB. It is now clear, however, that it can be better described as an immune “specialized” site, in which the bidirectional exchange of immune cells with the circulation is fundamental for the maintenance of integrity and functions [73, 74]. In addition, resident microglia can adopt active phagocytic behaviour and release proinflammatory factors upon brain injury [75, 76]. In the context of gliomas, however, glioma-associated microglia and macrophages (GAMs) seem to loss the ability to induce antitumour immune response and to switch instead to a tumour-promoting, immunosuppressive phenotype. The inducers of this behavioural transition are glioma-derived factors, among which are TGF*β*, interleukin- (IL-) 4, IL-6, IL-10, and prostaglandin PGE-2 [76, 77]. At the same time, glioma cells seem to be also able to recruit from the circulation other immature myeloid cells, which also adopt an immune suppressive behaviour, as soon as they start interacting with the cancer site [76]. The cellular and molecular bases for these phenotypic modifications of immune cells, which infiltrate the tumour, are not completely understood but certainly depend on an intense cross talk among glioma and surrounding cells, forced to conform to the signals received and involve at least in part EVs [78].

Among the proteins released not only from glioma cells, but also from GAMs, an important class is represented by the matrix metalloproteases (MMPs) [79–81], especially MMP-2 and MMP-9 [76, 82, 83]. These enzymes can mediate degradation of the extracellular matrix, thus promoting invasion of the surrounding healthy tissues. In line with this idea, microvesicles shed in culture by oligodendroglioma cells were, for instance, shown to contain a TIMP3-sensitive “aggrecanase” activity, which could allow cellular invasion of aggrecan-rich extracellular matrices [23].

Importantly, migration of glioma cells throughout the brain is also guided by the brain vessels, and cancer growth is associated with angiogenesis [69]. Proliferation of BCECs, to produce the new vessels, causes disruption of the tight junctions and generalized fragility of the BBB, which becomes leaky, thus creating the conditions for the vasogenic brain edema, the most serious clinical complication of glioblastoma [69]. Edema and glioma progression have been also correlated with altered expression of different isoforms of aquaporins (AQPs), a family of water channels of the plasma membrane [84], which are upregulated in brain cancer [85–89]. Since in other systems both proteins of the tight junctions [90] and AQPs [91, 92] have been reported to enter exosomes, a new approach to the study of glioma-linked edema could be the search for these proteins in EVs released from brain cancers, with the additional aim of identifying further diagnostic tools. Actually, variations in the number, and possibly in the function, of EVs circulating in peripheral blood have been reported in brain tumors [93].

Importantly, aggressiveness of gliomas has been correlated with hypoxia, which should be the main inducer of both necrosis and BBB alteration. Hypoxia-dependent intercellular signalling, induced by growth factors, such as VEGF, at least in part secreted via EVs [94], stimulates not only BCECs proliferation and angiogenesis, but also activation of the coagulation system, responsible for vascular thrombosis [95]. Microvesicles have been known to be associated with coagulation since the 1940s because they expose PS, a negatively charged phospholipid, which can allow recruitment of calcium ions and active coagulation factors. Actually, tumour-derived vesicles have an even higher coagulation potential because they carry TF (tissue factor), an initiating coagulation factor [78].

An additional serious problem, posed by brain tumours, is the cancer-induced neuronal cell death and neurodegeneration. These events are associated with cytotoxic edema [69, 96]. Again, extracellular vesicles are probably involved. For example, G26/24 oligodendroglioma cells release EVs that, when added to primary cultures of rat cortical neurons, inhibit neurite outgrowth and induce apoptosis in about 75% of the cells [16]. The same amount of EVs induces apoptosis in only 40% of astrocytes [97]. In line with these observations, vesicles released by G26/24 cells were found to contain extracellular proapoptotic ligands, such as FasL and TRAIL, which could cooperate in inducing brain cell death. As already mentioned, these effects depend, at least in part, on the horizontal transfer of proteins, mediated by the vesicles, from tumour to normal brain cells [16, 97].

EVs are probably also involved in expelling from cancer cells regulatory proteins. G26/24 release, for example, vesicles which carry on the differentiation-specific H1° histone variant, thus eliminating a protein otherwise able to counteract proliferation [37].

Perhaps the most intriguing property of EVs is that they contain various classes of nucleic acids and are therefore able to exert an effect on the translational profile of normal cells present in their environment [78, 98, 99]. Transfer of DNA among cells is an evolutionary conserved process: bacterial vesicles contain DNA encoding virulence genes, which can be transferred into other bacteria and then expressed [72]. EVs released from eukaryotic cells carry both DNA and RNA. For example, brain tumours release EVs which contain c-Myc as well as high levels of retrotransposon RNA transcripts, such as those for LINE-1 and Alu elements; these transposable elements are then transferred to normal cells [100]. The presence of noncoding RNAs (ncRNAs) in EVs is actually one of the more fascinating topics in the field, since at least some of these RNAs have been suggested to be involved in epigenetic regulation of gene expression [101] and have thus the potential to induce profound modifications of recipient cells attitudes.

Intriguingly, exosomes released from astrocytes and glioma cells also contain mitochondrial DNA [102].

The collection of glioma-derived mRNAs, transferred into recipient cells via EVs, is actually highly complex and includes a variety of transcripts associated with proliferation, immune repression, and tissue invasion [103]; these transcripts are representative of almost all the transcriptomes of the producing cells; however, some transcripts are clearly enriched in EVs [98], thus suggesting existence of mechanisms, such as the presence of specific “zip code-like” sequences in the untranslated regions of the target passenger mRNAs [104], which allow selective sorting and/or stabilization of given mRNAs in EVs. Interestingly, many transcripts are common among EVs released from different cancer cell types [98].

Among EV-carried RNAs, a special class, which is attracting much attention, is represented by microRNAs [98]. Ten years ago, a microarray analysis allowed, for the first time, identification in glioblastoma, by two different laboratories, of aberrant miRNAs; one of which (miR21) was in particular shown to act as an antiapoptotic factor (for a review see [105]). More recently, a genome-wide miRNA expression profile allowed identification of 55 upregulated and 29 downregulated miRNAs in malignant gliomas, at the same time suggesting that a group of 23 miRNAs could represent a sort of signature for GBM able to distinguish it even from anaplastic astrocytoma [106]. Although more studies are requested to univocally combine all the results described in the last ten years, a few miRNAs have indeed the potential to contribute to GBM [105, 107].

By performing microRNA PCR array, Camacho et al. also found a difference among brain metastatic (BM) and nonbrain metastatic tumor-derived exosomes; in particular, one upregulated (miR-210) and two downregulated (miR-19a and miR-29c) miRNAs were identified in BM versus non-BM exosomes [108].

The presence of both the proteins and RNAs discussed above in glioma-derived EVs, combined with the fact that these EVs are also present in the circulation of patients with glioma, while they almost disappear after tumor removal [103], suggests that these vesicles could be used as a liquid biopsy of cancer and reflect the disease grade [109–111]. A specific biomarker for glioblastoma is, for example, the already mentioned EGFRvIII, a mutated EGF receptor which lacks the extracellular domain, and triggers a “constitutively on” signal transduction pathway. This protein is found both in tumor cells and in shed vesicles [71, 112].

Recently, the ability of tumor cells to cause damage not only by acting directly on normal cells but also by altering their extracellular environment has been also emerging [113].

Finally, it should be mentioned that exosomes may protect tumor cells from accumulating drugs, thus accounting, at least in part, for drug/multidrug resistance [78, 114]. For example, P-glycoprotein, member of the ATP binding cassette superfamily, and one of the most important drug transporters, has been shown to be exchanged among cells via EVs [115].

## 4. Neurodegenerative Diseases

As in the case of brain tumors, variations in the amount of EVs circulating in peripheral blood have been also reported in several nervous system diseases, such as Alzheimer's disease [116], dementia [117], epilepsy [118], stroke [119], and traumatic brain injury [120]. The association between EV increase and multiple sclerosis is also well documented (as reviewed in [121, 122]), although the exact role of shed vesicles in disease progression is still unclear: it seems that they contain metalloproteinases damaging the blood-brain barrier, as well as factors involved in propagation of neuroinflammation; on the other hand, they seem to promote maturation and migration of oligodendrocyte precursor cells, necessary for repair of the damaged axons [121].

EVs can be implicated in neurodegenerative diseases also because they can deliver toxic proteins such as prions (PrPsc) [123–125], alpha-synuclein [126, 127], amyloid precursor protein (APP) or *β*-amyloid peptides [128–133], phosphorylated Tau [134], and SOD1 [135].

Accumulation of aggregates of abnormal proteins has indeed emerged as a common mechanism for most human neurodegenerative diseases, including Alzheimer's disease, Parkinson's disease, frontotemporal dementias, and amyotrophic lateral sclerosis. All these diseases should propagate through the brain via prion-like intercellular induction of protein misfolding [136, 137]. Neurons are peculiar cells in that they are postmitotic and cannot self-renew to clear abnormally folded/accumulated proteins; thus they accumulate protein folding errors, especially into aggregation-prone proteins, throughout their life-span [138]. Defects in protein degradation by the proteasome-depending pathway, because of abnormal activity of ubiquitinating/deubiquitinating enzymes, molecular chaperones, and protein hydrolases, can severely affect cell health and survival.

One of the most studied examples of the damaging effects of protein aggregation in neurons is given by the microtubule-associated protein Tau. An increase in Tau concentration, hyperphosphorylation, and aggregation seems to be the principal agent in the transmission and spreading of tauopathies, among which is Alzheimer's disease. Intracellular accumulation of aggregated Tau has been considered over time the main source of its toxicity. However, Tau added from the extracellular side is still toxic to neuronal cells [139]. For a long time, extracellular tau was believed to come out from lysed dead neurons. More recently, however, growing evidence suggested that extracellular Tau in AD brain (and CSF) is very likely due to active secretion [140–143].

A nontransgenic lower vertebrate tauopathy model (the lamprey ABC model) has been used to express full-length wild type and mutant human Tau isoforms in identified neurons, thus allowing localization of toxic Tau sources. Thanks to this model system, Tau was found to be secreted before the onset of neuronal degeneration and to be transferred among neurons, thus spreading in a disease-specific pattern to the brain and playing a major role in pathogenesis [144, 145]. Association of Tau with exosomes suggests that extracellular vesicles is at least one of the routes for active interneuronal transfer of toxic protein [140]. Interestingly, it has been also found that Tau can interact with signaling components localized to the plasma membrane, such as the Src-family of nonreceptor tyrosine kinases [141, 145, 146]. This finding suggests that extracellular signaling might have an effect on Tau sorting to extracellular vesicles and spreading throughout the brain.

Similar to Tau protein, in synucleinopathies, such as Parkinson's disease, SNCA/*α*-synuclein can be released by neural cells into the extracellular space by EV shedding [125, 126]. It has been reported that SNCA/*α*-synuclein is released in two main forms: through exosomes, which contain low-aggregated proteins, and through MVs, which contain high-aggregated proteins. It seems that, while intracellular SNCA aggregation has probably a protective role, released SNCA is toxic, most probably spreading the disease throughout the brain; the toxic effect is highly enhanced when autophagy-lysosomal pathway is inhibited [147].

Involvement of EVs in the pathological spreading of toxic proteins has been confirmed by the presence of vesicles in the CSF. MVs derived from all the major types of neural cells are already detectable in both rodent and human CSF, under normal conditions. In the inflamed brain, in cases of multiple sclerosis in humans and experimental autoimmune encephalomyelitis (EAE) in mice, however, the amount of MVs increases dramatically depending on disease severity and the extent of microglia activation [148]. Exosomes enriched in prion proteins have been isolated from both ovine CSF [149] and human CSF [150].

In the late 1990s, human Tau was already evidenced in CSF of early stage patients with Alzheimer's disease [151, 152]. More recently, phosphorylated Tau has been found in exosomes, and this is a peculiar feature of AD with respect to both normal aging and other neurodegenerative diseases [141]. The early presence of exosome-associated Tau in CSF is of interest for at least two reasons: (1) it is a further indication of an active secretion of Tau from neurons (discussed in the previous section); (2) it could offer a powerful and noninvasive instrument for early AD diagnosis [138].

Given their potential interest in both diagnosis and basic research, EVs from the CSF have been the subjects of several studies. In particular, many efforts have been devoted to proteomic profiling of CSF exosomes [150, 153]. These analyses demonstrated the presence in CSF vesicles, among other proteins, of Alix, syntenin-1, tetraspanins, heat shock proteins, Rab proteins, transcription factors, MHC antigens, integrin alpha-M, the receptor-type tyrosine-protein phosphatase C, enolase 2, the dihydropyrimidinase-related protein 2, and the vesicle-associated membrane protein 2 (VAMP2) [150].

From all the discussed studies, it is evident that EVs, if taken up from cells different from the producer ones, have the potential to promote deep modifications of properties and behavior of the recipient cells.

## 5. Concluding Remarks

In conclusion, extracellular vesicles seem to play an important role in coordinating intercellular communication in brain. Production of EVs is probably a dynamic process which can undergo both quantitative and qualitative modifications depending on neuronal activity, metabolic state, and perhaps membrane trafficking rate. In pathological conditions, such as cancer as well as neurodegeneration, EV production and release seem to be potentiated and allow secretion into the extracellular environment of proteins, RNAs, and lipids, which can horizontally transfer pathological features to the surrounding cells. The presence of disease-specific proteins and RNAs in EVs, which can also reach the patient CSF, could offer a powerful way for early detection of pathology and is certainly worth of further analyses. Finally, EVs also deserve attention as potential drug carriers, theoretically able to cross the blood-brain barrier.
